# Sex Differences in Cancer Immunotherapy—Clinical Evidence and Mechanisms With a Focus on NSCLC

**DOI:** 10.1111/imr.70113

**Published:** 2026-02-27

**Authors:** Eva Krieghoff‐Henning, Balagopal Pai, Maike Collienne, Isabel Ben‐Batalla, Sonja Loges

**Affiliations:** ^1^ Department of Personalized Oncology University Hospital Mannheim, Heidelberg University Mannheim Germany; ^2^ Division of Personalized Medical Oncology German Cancer Research Center (DKFZ) Heidelberg Germany; ^3^ DKFZ Hector Cancer Institute at the University Medical Center Mannheim Mannheim Germany; ^4^ Division of Translational Medical Oncology German Cancer Research Center (DKFZ) Heidelberg Germany; ^5^ National Center for Tumor Diseases (NCT), NCT Heidelberg A Partnership Between DKFZ and Heidelberg University Hospital Heidelberg Germany; ^6^ Department of Physiology, Center for Research in Molecular Medicine and Chronic Diseases (CiMUS) University of Santiago de Compostela Santiago de Compostela Spain; ^7^ German Center for Lung Research (DZL) German Cancer Consortium (DKTK) Heidelberg Germany

**Keywords:** immune checkpoint inhibitor, immunotherapy, non‐small cell lung cancer/NSCLC, sex differences, testosterone, tumor microenvironment/TME

## Abstract

Accumulating evidence suggests that the immune system shows subtle but relevant differences between men and women. These differences may have an impact on cancer development and TME composition as well as responses to and adverse events elicited by immunotherapies. Several, albeit not all, clinical trials indicate a greater benefit from mono‐immunotherapies over chemotherapies for male patients than for female patients, especially in non‐small cell lung cancer and melanoma. Vice versa, female patients might benefit more from chemo‐immunotherapies. In human as well as animal models, sex differences in cancer microenvironment composition were described, with partially divergent results. Sex‐specific factors such as the levels of hormones, in particular testosterone and estrogen, or X‐ or Y‐chromosome associated genes are likely to drive the observed differences, but are often confounded by external influences such as smoking behavior, diet, or UV exposure. Therefore, large clinical and mechanistic knowledge gaps remain regarding the influence of sex on cancer immunotherapies and strategies to optimize response in either sex. More clinical as well as experimental research in this field is required to close these knowledge gaps, and clinical trials should include large enough groups of male and/or female patients to allow robust sex‐specific analyses.

## 1 Main

Although it may appear obvious that the known physiological differences that exist between men and women may also influence health overall, disease susceptibility and treatment outcomes, the field of “gender medicine” is still in its relative infancy, particularly with respect to cancer that is not exclusive to one sex. In this review, we revisit the available data on potential differences between the sexes with a focus on non‐small cell lung cancer (NSCLC) and immune checkpoint inhibitor therapy, both from a clinical and a more mechanistic basic research perspective. In line with common practice, we refer to patients' sex when we refer to “intrinsic”, physiologic, sex‐chromosomally driven factors that differentiate between biological male and female individuals, while we refer to patients' gender when non‐biological factors such as lifestyle differences are considered.

## 2 Sex‐Specific Differences in Cancer Incidence

For cancer overall, men used to have a substantially higher cancer incidence than women, with a few notable exceptions such as thyroid and gallbladder cancer [1, 2]. Disparities in male versus female cancer incidence may also occur in rare cancers that have no known external risk factors, such as desmoplastic small round cell tumor (DSRCT), which often occurs early in life and has a strong male predominance of at least 4:1 [3]. However, the overall cancer incidence gap between women and men is narrowing in countries such as the US [4] or Germany [5]. This also includes the two most prominent cancer types that the initial immunotherapy trials focused on, non‐small cell lung cancer (NSCLC) and malignant melanoma. The changing patterns regarding cancer overall likely reflect associations of many of the predominant cancer types with lifestyle factors including, but extending beyond smoking, such as tanning behavior, UV exposure and protection, diet including alcohol consumption, (lack of) physical activity and overweight, suggesting that current clinical trials should not necessarily have an inherent sex/gender bias towards males due to incidence anymore. At present, large gender gaps regarding NSCLC incidence remain in countries where smoking patterns are still very divergent between genders, or have only recently begun to change [6]. In the US, women were reported to have surpassed the lung cancer incidence rate of males already in 2021. Patterns of melanoma incidence in men and women were also shown to change. Overall, melanoma incidence appears to be dropping again in the more recent birth cohorts, but more consistently in males than in females, especially with respect to trunk melanomas. Sex‐specific differences in the predominant melanoma localization still appear to persist, that is, a higher incidence of melanomas on the trunk for males and on the lower limbs for females [7]. In lung cancer, proportionally more non‐smoker's lung cancer and more driver mutation‐dependent lung cancer occur in women [8, 9].

This suggests that cancers may differ qualitatively between men and women also for common cancers with lifestyle‐associated external risk factors, and differences in incidence of rarer, more lifestyle‐independent cancer entities may persist. Moreover, the kinetics of cancer development may differ, with some cancers occurring more frequently in young women versus young men, but a reversed relationship in older individuals, or vice versa (e.g., for melanomas [4]).

## 3 Sex‐Specific Differences in Clinical Trial Participation

Historically, females have always been underrepresented in cancer trials [10, 11, 12]. As discussed in more detail below, this can be partially explained by a higher incidence of many non‐reproductive cancers in men. However, the proportion of women in cancer trials was still found to be lower than what would be expected due to this difference in incidence. Contributing reasons may be varied, for example, a reluctance to enroll premenopausal women in trials with new and potentially embryotoxic and/or fertility‐reducing treatments, greater child‐ and family care responsibilities, sociocultural barriers, etc. [12]. For immunotherapy trials, the observation that women are overall more prone to autoimmune diseases (see below) and a perceived high likelihood that immune‐activating therapies would reduce the immune tolerance required for pregnancy [13] may have exacerbated this problem. For NSCLC, the higher proportion of driver mutation with a negative impact on immunotherapy responses might also have contributed to this discrepancy in trials that were conducted after this association had been recognized. In consequence, effects observed in clinical trials were overall more strongly driven by the male patients included in these trials.

The underrepresentation of females was likely not considered a major problem because the general expectation was that drug responses were generally comparable between men and women. Thus, sex‐specific analyses were often not conducted. However, starting in the Nineties of the last century, institutions such as the FDA realized that female underrepresentation might be a problem and began to issue recommendations to equalize the participation of both genders/sexes in clinical trials, starting already in 1993, and have even proceeded to provide very concrete suggestions of gender‐specific factors such as the influence of the menstrual cycle or exogenous hormones on pharmacokinetics [14, 15].

## 4 Sex Differences in Immunotherapy Outcomes—The First Meta‐Analysis “Wave”

**FIGURE 1:**
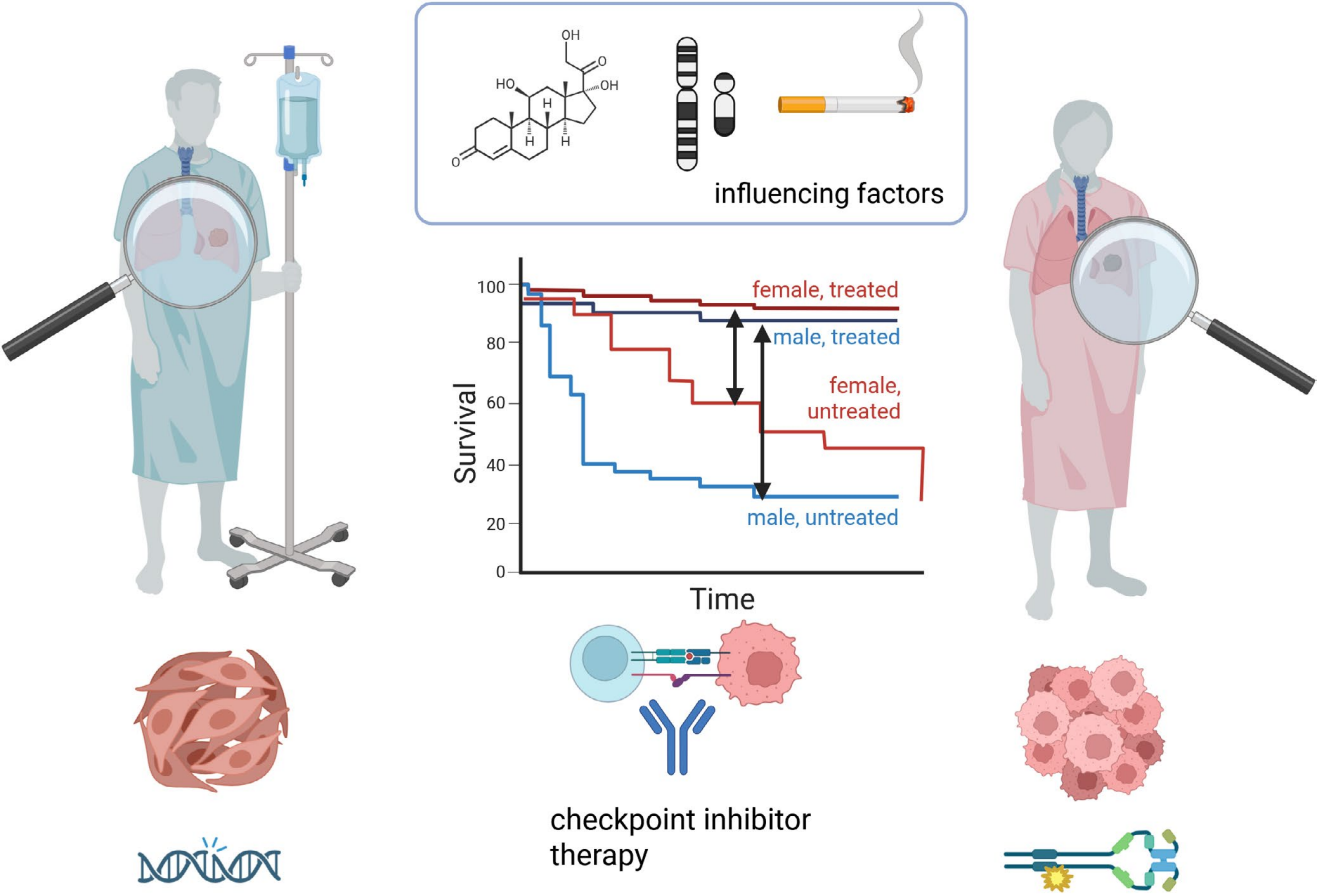
Sex differences in lung cancer immunotherapy—overview. Studies indicate that lung tumors in male versus female patients differ in their etiology, composition and baseline prognosis, and may respond differently to immunotherapies, with a greater improvement in male patients under checkpoint inhibitor therapy. Figure created using BioRender.

In the field of immuno‐oncology in particular, in 2018/19, first meta‐analyses encompassing immune checkpoint inhibitor trials, most prominently publications from the Goldhirsch group [16, 17] suggested that men might profit more from immune checkpoint inhibition relative to the previous standard treatment chemotherapy, whereas women might benefit more from a combination of immune checkpoint inhibitors with chemotherapy (Figure 1). In the 2018 publication, 20 RCTs across diverse cancer types that were reported to respond to immune checkpoint inhibitors (7 melanoma, 6 NSCLC, 3 head and neck SCC, 1 each of SCLC, mesothelioma, RCC, urothelial carcinoma, GEJ cancer) were included, yielding an overall difference in overall survival hazard ratio (HR) in favor of immunotherapy of 0.72 in men and 0.86 in women. In these trials, only the CTLA‐4 inhibitor ipilimumab was combined with chemotherapy, whereas all other trials compared checkpoint inhibitor monotherapy to standard therapies in the respective cancer entity. In the follow‐up publication, Conforti et al. focused on lung cancer and trials where checkpoint inhibitors were combined with chemotherapy and compared with chemotherapy alone, this time pointing to a larger benefit for females (OS HR of 0.48 for women versus 0.76 for males) [17]. In other meta‐analyses with only partially overlapping studies, across all studies, trends for differential outcomes between men and women were less clear and not statistically significant [18] (IO versus non‐IO *p* = 0.33, IO plus non‐IO versus non‐IO, *p* = 0.55; [19], male HR 0.69, female HR 0.70 for checkpoint inhibitors versus docetaxel). These discrepant findings have triggered a wave of investigations into sex and gender differences in immunotherapy and other clinical trials in the following years (see Figure 1).

## 5 Sex Differences in Immunotherapy Trials for Advanced NSCLC

As mentioned above, in many of the trials investigating immune checkpoint inhibitors for the treatment of advanced cancers, potential differences in benefit according to sex were found. Prominent examples were the KEYNOTE‐024 trial and the EMPOWER‐Lung 1 trial in NSCLC, or the CheckMate 066 study in melanoma [20, 21, 22, 23].

In KEYNOTE‐024, NSCLC patients received either the PD‐1‐directed checkpoint inhibitor pembrolizumab or platinum‐based chemotherapy as first‐line treatment in the palliative setting [20, 23]. 61% of the participants were male. In the 2016 analysis, the hazard ratio (HR) for progression or death was 0.39 (95% CI 0.26–0.58) in males, and 0.75 (95% CI 0.46–1.21) in females, and in the 2019 analysis, HR for OS was 0.54 (0.36–0.79) in males versus 0.95 (0.56–1.62) in females. Thus, male patients appeared to derive a much greater benefit than female patients from replacing the standard chemotherapy with a checkpoint inhibitor. In the EMPOWER‐Lung 1, where cemiplimab was used instead of pembrolizumab in a similar setting, women also derived little or no overall survival benefit from immunotherapy instead of standard chemotherapy [21, 24]. In the cohort with PD‐L1 levels ≥ 50%, the HR for OS was 0.50 (95% CI 0.36–0.69) in males compared to 1.11 (95% CI 0.49–2.52) in females, with a similar distribution after 5 years (0.521 vs. 0.918). Of note, the proportion of female patients in this trial was particularly low, with only 15% (*n* = 104) women in the entire cohort. In the CheckMate 066 trial, 246 male and 172 female patients with advanced melanoma received the PD‐1‐directed checkpoint inhibitor nivolumab or palliative chemotherapy with Dacarbazine [22]. Again, the comparison revealed a larger benefit for male patients, with an unstratified HR for death of 0.34 (95% CI 0.22–0.54) in male versus 0.56 (95% CI 0.33–0.95) in female patients. In other trials, settings and/or cancer entities, these effects were less clear. For instance, in the Keynote–010 trial, where NSCLC patients received either pembrolizumab or docetaxel as second‐line treatment, there was a minimal numerical advantage in long‐term survival for female patients (HR for OS: 0.71 (95% CI 0.60–0.86) in males, 0.66 (95% CI 0.53–0.84) in females) [25]. Other, similar trials were the KEYNOTE‐042 [26], IMpower110 [27], CheckMate 026 [28], CheckMate 017 [29], KEYNOTE‐010 [30] or CheckMate 057 [31]. Results are summarized in Table 1.

**TABLE 1:** Overview of studies with non‐small cell lung cancer (NSCLC) patients receiving checkpoint inhibitors with or without chemotherapy.

Study	Tumor entity	Checkpoint inhibitor(s)	Compared against	Setting	Males/females	Outcome details	Reference(s)
*Checkpoint inhibition only, palliative*							
KEYNOTE‐024 (NCT02142738)	NSCLC, PD‐L1 ≥ 50%, stage IV	Pembrolizumab	Chemotherapy (platinum‐based)	Palliative (1st line)	187 (61%)/118 (39%)	HR for progression or death: 0.39 (95% CI 0.26–0.58) in males, 0.75 (95% CI 0.46–1.21) in females; HR for OS: 0.54 (0.36–0.79) in males, 0.95 (0.56–1.62) in females	Reck et al. [20, 23]
EMPOWER‐Lung 1 (NCT03088540)	NSCLC, locally advanced or metastatic	Cemiplimab	Chemotherapy (platinum‐based)	Palliative (1st line)	479 (85%) vs. 84 (15%) in the ≥ 50% PD‐L1 group	In the ≥ 50% PD‐L1 group, median 11 months follow‐up: HR for PFS 0.50 (0.40–0.64) in males, 0.79 (0.43–1.46) in females; HR for OS: 0.50 (0.36–0.69) in males, 1.11 (0.49–2.52) in females with PD‐L1 ≥ 50% 5‐year follow‐up: HR for OS 0.521 (0.417–0.650) in males, 0.918 (0.520–1.620) in females	Sezer et al., Kilickap et al. [21, 24]
KEYNOTE‐042 (NCT02220894)	NSCLC, locally advanced or metastatic, PD‐L1 TPS of 1% or more	Pembrolizumab	Chemotherapy (platinum‐based)	Palliative (1st line)	TPS ≥ 50% group: 415/184	TPS ≥ 50% group: HR for OS: 0.68 (0.53–0.88) in males, 0.78 (0.53–1.15) in females	Mok et al. [26]
IMpower110 (NCT02409342)	NSCLC, metastatic, PD‐L1 of at least 1% on tumor or immune cells	Atezolizumab	Chemotherapy (platinum‐based)	Palliative (1st line)	PD‐L1 high group: 143 (70%)/62 (30%)	PD‐L1 high group: HR for death: 0.57 (0.35–0.93) in males, 0.69 (0.34–1.39) in females	Herbst et al. [27]
CheckMate 026 (NCT02041533)	NSCLC, stage IV or recurrent, (TPS of at least 1%, for primary efficacy at least 5%, no EGFR or Alk mutations)	Nivolumab	Chemotherapy (platinum‐based)	Palliative (1st line)	332 (61%)/209 (39%)	Unstratified HR for PFS 1.05 (0.81–1.37) in males, 1.36 (0.98–1.90) in females; unstratified HR for OS: 0.97 (0.74–1.26) in males, 1.15 (0.79–1.66) in females	Carbone et al. [28]
CheckMate 017 (NCT01642004)	NSCLC (squamous), stage IIIB or IV	Nivolumab	Chemotherapy (docetaxel)	Palliative (2nd line, after progression on platinum doublet)	208 (76%)/64 (24%)	Unstratified HR for PFS 0.63 (0.46–0.85) in males, 0.71 (0.40–1.26) in females; Unstratified HR for OS: 0.57 (0.41–0.78) in males, 0.67 (0.36–1.25) in females	Brahmer et al. [29]
KEYNOTE‐010 (NCT01905657)	NSCLC, advanced, TPS of at least 1%	Pembrolizumab	Chemotherapy (docetaxel)	Palliative (2nd or further line)	634 (61%)/399 (39%)	1 year‐analysis: HR for OS 0.65 (0.52–0.81) in males, 0.69 (0.51–0.94) in females; 5‐year analysis, HR for OS: 0.71 (0.60–0.86) in males, 0.66 (0.53–0.84) in females	Herbst et al. [30]
CheckMate 057 (NCT01673867)	NSCLC (non‐squamous), stage IIIB, IV or recurrent	Nivolumab	Chemotherapy (docetaxel)	Palliative (2nd line, after platinum doublet)	319 (55%)/263 (45%)	Unstratified HR for OS: 0.73 (0.56–0.96) in males, 0.78 (0.58–1.04) in females	Borghaei et al. [31]
*ICI + chemo, palliative*							
IMpower150 (NCT02366143)	NSCLC (non‐squamous), metastatic	Atezolizumab/chemotherapy (+/− bev) (ACP and ABCP)	Platinum‐based (chemotherapy + bevacizumab) (BCP)	Palliative (chemotherapy‐naive)	425 (61%)/267 (39%) in the INTENTION‐TO‐TREAT wt group	ABCP vs. BCP, initial analysis: HR for PFS 0.55 (8.4 vs. 6.8 month) in males, 0.73 (8.2 vs. 6.8 month) in females in the INTENTION‐TO‐TREAT wt population; final analysis, HR for OS 0.72 (0.58–0.90) in males, 0.92 (0.70–1.22) in females	Socinski et al. [33]
IMpower130 (NCT02367781)	NSCLC (non‐squamous), metastatic	Atezolizumab/chemo	Platinum‐based chemo	Palliative (chemotherapy‐naive)	INTENTION‐TO‐TREAT wt group: 400 (59%)/279 (41%)	INTENTION‐TO‐TREAT wt group: HR for OS 0.87 (0.66–1.15) in males, 0.66 (0.46–0.93) in females	West et al. [32]
CheckMate 9LA (NCT03215706)	NSCLC, stage IV or recurrent	Nivolumab/ipilimumab plus chemotherapy (2 cycles)	Chemotherapy only	Palliative (first‐line)	504 (70%)/215 (30%)	Unstratified HR for death: 0.66 (0.53–0.82) in males, 0.68 (0.47–1.00) in females	Paz‐Ares et al. [34]
RATIONALE 307 (NCT03594747)	NSCLC (squamous), stage IIIB or IV	Tislelizumab/chemotherapy (either platinum‐based or nab‐paclitaxel)	Chemotherapy (either platinum‐based or nab‐paclitaxel)	Palliative (first‐line)	330 (91.7%)/30 (8.3%)	Initial analysis: HR for PD or death 0.53 (95% CI 0.37–0.76) in males, 0.53 (95% CI 0.17–1.61) in females for platinum‐based chemo; 0.50 (0.35–0.71) in males, 0.36 (95% CI 0.09–1.47) in females for nab‐paclitaxel; final analysis: HR for PFS 0.49 (0.36–0.69) in males, 0.34 (0.12–0.99) in females for platinum‐based chemo, 0.46 (0.33–0.64) in males, 0.46 (0.13–1.61) in females for nab‐paclitaxel	Wang et al. [35]
RATIONALE 304 (NCT03663205)	NSCLC (non‐squamous), locally advanced or metastatic	Tislelizumab/chemotherapy (platinum‐based)	Chemotherapy only (platinum‐based)	Palliative (first‐line)	247 (74%)/87 (26%)	Unstratified HR for PFS 0.53 (0.37–0.75) in males, 0.85 (0.49–1.48) in females, unstratified HR for OS 0.84 (0.57–1.25) in males, 1.02 (0.48–2.18) in females	Lu et al. [36]
KEYNOTE‐189 (NCT02578680)	NSCLC (non‐squamous), metastatic	Pembrolizumab/chemotherapy (platinum‐based)	Chemotherapy only (platinum‐based)	Palliative (first‐line)	363 (59%)/253 (41%)	Initial analysis: HR for OS 0.70 (0.50–0.99) in males, 0.29 (0.19–0.44) in females; final analysis: HR for PFS 0.58 (0.46–0.74) in males, 0.39 (0.29–0.52) in females HR for OS 0.74 (0.56–0.96) in males, 0.41 (0.30–0.56) in females	Rodríguez‐Abreu et al. [37]
KEYNOTE‐407 (NCT02775435)	NSCLC (squamous), metastatic	Pembrolizumab/chemotherapy (platinum‐based)	Chemotherapy only (platinum‐based)	Palliative (first‐line)	455 (81%)/104 (19%)	HR for Death 0.69 (0.51–0.94) in males, 0.42 (0.22–0.81) in females; HR for disease progression or death 0.58 (0.46–0.73) in males, 0.49 (0.30–0.81) in females	Paz‐Ares et al. [38]
IMpower131 (NCT02367794)	NSCLC (squamous), stage IV	Atezolizumab/chemotherapy (platinum‐based, carboplatin plus either paclitaxel (ACP) or nab‐paclitaxel (ACnP))	Chemotherapy only (platinum‐based, carboplatin with nab‐paclitaxel (CnP))	Palliative (first‐line/chemotherapy naive)	ACnP and CnP: 557 (82%)/126 (18%)	Unstratified HR for OS in INTENTION‐TO‐TREAT at final analysis, ACnP vs. CnP: 0.91 (0.75–1.12) in males, 0.68 (0.44–1.04) in females; unstratified HR for PFS in INTENTION‐TO‐TREAT at primary analysis: 0.71 (0.59–0.85) in males, 0.66 (0.45–0.97) in females	Jotte et al. [39]
IMpower132 (NCT02657434)	NSCLC (non‐squamous), stage IV	Atezolizumab/chemotherapy (platinum‐based)	Chemotherapy only (platinum‐based)	Palliative (first‐line/chemotherapy naive)	Primary analysis: 384 (66.4%)/178 (33.6%)	Primary analysis: HR for PFS 0.64 (0.51–0.79) in males, 0.51 (0.36–0.71), HR for OS 0.93 (0.73–1.18) in males, 0.76 (0.54–1.09) in females	Nishio et al. [40]
PACIFIC (NCT02125461)	NSCLC (non‐progressive after chemoradiotherapy), locally advanced unresectable	Durvalumab (maintenance after chemoradiotherapy)	Placebo (maintenance after chemoradiotherapy)	Palliative (as maintenance after chemoradiotherapy)	500 (70.1%)/213 (29.9%)	Unstratified HR for Death: 0.78 (0.59–1.03) in males, 0.46 (0.30–0.73) in females; unstratified HR for disease progression or death 0.56 (0.44–0.71) in males, 0.54 (0.37–0.79) in females; 3‐year update: unstratified HR for death 0.74 (0.57–0.95) in males, 0.50 (0.35–0.81) in females; HR for OS INTENTION‐TO‐TREAT 5‐year update: 1.27 for males compared to females	Antonia et al., Gray et al., Spigel et al. [41, 42, 43]
OAK (NCT02008227)	NSCLC, stage IIIB or IV	Atezolizumab	Docetaxel	Palliative (chemotherapy pre‐treated)	520 (61%)/330 (39%)	HR for OS: 0.79 (0.64–0.97) in males, 0.64 (0.49–0.85) in females	Rittmeyer et al. [44]
POPLAR (NCT01903993)	NSCLC, advanced	Atezolizumab	Docetaxel	Palliative (chemotherapy pre‐treated)	169 (59%)/118 (41%)	n.a.	Fehrenbacher et al., Mazieres et al. [45, 46]
*Early disease setting*							
PEARLS/KEYNOTE‐091 (NCT02504372)	NSCLC, stage IB–IIIA	Pembrolizumab	Placebo	Adjuvant (mostly after chemo)	804 (68%)/373 (32%) in the INTENTION‐TO‐TREAT population, 237 (71%)/96 (29%) in the TPS ≥ 50% population	HR for DFS: 0.81 (0.65–1.01) in males, 0.73 (0.54–1.00) in females	O'Brien et al. [47]
KEYNOTE‐671	NSCLC, resectable stage II, IIIA or IIIB	Pembrolizumab plus chemotherapy (platinum‐based)	Placebo plus chemotherapy (platinum‐based)	Adjuvant/neoadjuvant	563 (71%)/234 (29%)	HR for EFS 0.63 (0.49–0.80) in males, 0.44 (0.28–0.68) in females	Wakelee et al. [53]
IMpower010	NSCLC, stage II—IIIA	Chemotherapy followed by atezolizumab	Chemotherapy (various) followed by BSC	Adjuvant/neoadjuvant	589 (67%)/293 (33%) overall	HR for DFS: PD‐L1 ≥ 1% 0.69 (0.48–0.99) in males, 0.61 (0.38–0.97) in females; entire population 0.76 (0.59–0.99) in males, 0.80 (0.57–1.13) in females	Felip et al. [48]
BR.31	NSCLC, Stage IB ‐IIIA, PD‐L1 TC positive	Optional adjuvant chemotherapy followed by durvalumab	Optional adjuvant chemotherapy plus placebo	Adjuvant (after complete resection)	943 (66%)/481 (34%)	HR for DFS 0.92 (0.75–1.14) in males, 0.83 (0.61–1.12) in females	Goss et al. [51]
NADIM II	NSCLC, resectable stage III	Neoadjuvant chemotherapy (carboplatin/paclitaxel) plus nivolumab, followed by adjuvant nivolumab	n.a. (single arm)	Adjuvant (after complete resection)	52 (61%)/34 (39%)	Pathological complete response rate 30.6% with nivolumab vs. 0% without in males (unweighted difference 30.6%), 47.6% with nivolumab vs. 15.4% without in females (unweighted difference 32.2%)	Provencio et al. [54]
LCMC3	NSCLC, Stage IB through IIIB	Chemotherapy followed by atezolizumab (+/− radiotherapy)	n.a. (single arm)	Adjuvant	88 (49%)/93 (51%)	Major pathological response rate: 22/68 females (32.4%), 7/69 males (10.1%), ratio females vs. males 4.2, *p* = 0.002	Chaft et al. [52]
AEGEAN	NSCLC, Stage IIA through III‐B w/o EGFR and Alk mutations	Platinum‐based chemotherapy plus durvalumab	Platinum‐based chemotherapy plus placebo	Neoadjuvant	530 (72%)/210 (28%)	HR for EFS 0.61 (0.44–0.82) in males, 0.95 (0.58–1.56) in females	Heymach et al. [49]
CheckMate‐77 T	NSCLC, Stage IIA through III‐B w/o EGFR and Alk mutations	Chemotherapy plus nivolumab	Chemotherapy plus placebo	Neoadjuvant plus 1 year of adjuvant nivolumab	327 (71%)/134 (29%)	HR for EFS 0.53 (0.37–0.75) in males, 0.71 (0.41–1.20) in females; pathological complete rate 26.9 with nivolumab vs. 5% with chemotherapy in males, 21.0% with nivolumab vs. 4.2% with chemotherapy in females; unweighted difference 21.9% for males, 16.8% for females	Cascone et al. [50]
Neotorch	NSCLC, stage III	Chemotherapy (carboplatin/paclitaxel) plus toripalimab	Chemotherapy plus placebo	Neoadjuvant with adjuvant component	370 (92%)/34 (8%)	HR for EFS 0.38 (0.26–0.54) for males, 0.54 (0.15–1.80) for females	Lu et al. [55]
Rationale‐315	NSCLC, resectable stage II–III	Platinum‐based chemotherapy plus tislelizumab	Platinum‐based chemotherapy plus placebo	Neoadjuvant with adjuvant component	410 (91%)/43 (9%)	HR for EFS 0.57 (0.40–0.80) males, 0.68 (0.19–2.40) for females	Yue et al. [56]
NADIM	NSCLC, resectable stage III	Nivolumab plus platinum‐based chemotherapy	Chemotherapy	Neoadjuvant with adjuvant component	34 (74%)/12 (26%)	Initial analysis: HR for progression or death, 0.85 (0.18–4.09) for females relative to males; 5 years HR PFS 0.61 (0.13–2.83) for females compared to males; 5‐year analysis: HR for PFS 0.61 (0.13–2.83) in females relative to males, HR for OS: 0.32 (0.04–2.52) relative to males	Provencio et al.; Provencio et al. [58, 59]
CheckMate‐816 (NCT02998528)	NSCLC, resectable	Nivolumab plus chemo	Chemotherapy only	Neoadjuvant	255 (71%)/103 (29%)	HR for disease progression/recurrence/death: 0.68 (95% CI 0.47–0.98) in males, 0.46 (95% CI 0.22–0.96) in females; HR OS: 0.76 (0.53–1.09) for males, 0.52 (0.25–1.10) for females	Forde et al. [57]

Abbreviations: DFS, Disease‐free survival; EFS, Event‐free Survival; HR, Hazard Ratio; OS, Overall Survival; PFS, Progression‐free Survival.

## 6 Sex Differences in Chemo‐Immunotherapy Trials for Advanced NSCLC

Many trials have also investigated combinations of checkpoint inhibitors with chemotherapy in comparison with chemotherapy alone to treat NSCLC patients with advanced disease, both in the first line (IMpower 130 [32], IMpower 150 with chemotherapy‐naive patients [33], CheckMate 9LA with nivolumab plus ipilimumab [34], Rationale 307 and −304 [35, 36], KEYNOTE‐189 and ‐407 [37, 38]), chemotherapy‐naive patients that were allowed to have other pretreatments (IMpower 150, IMpower 131 and −132) [33, 39, 40], with a checkpoint inhibitor as maintenance after chemoradiotherapy (PACIFIC) [41, 42, 43], and in the chemotherapy‐pretreated setting (OAK, POPLAR, the latter without published sex‐specific analyses) [44, 45, 46]. Most of these trials showed at least a numerical advantage for females (e.g., KEYNOTE‐189, KEYNOTE‐407, IMpower 130, −131 and 132, OAK), but there were also trials showing no difference (Rationale 307, CheckMate 9LA) or even an advantage for male patients (Rationale 304). For a summary of NSCLC study settings and outcomes, see Table 1.

## 7 Sex Differences in Immunotherapy Trials for Early NSCLC

By now, there are also several trials in which immune checkpoint inhibitors were used for early‐stage NSCLC, such as the PEARLS/KEYNOTE‐091 [47], IMpower010 [48], AEGEAN [49], CheckMate‐77T [50], BR.31 [51], LCMC3 [52] or KEYNOTE‐671 [53] trial. In the adjuvant trials, the study treatment per se was an immune checkpoint inhibitor on its own (PEARLS [47], one option in BR.31 [51], NADIM II [54]), whereas in the neoadjuvant/perioperative trials (KEYNOTE‐671 [53], IMpower‐010 [48], AEGEAN [49], CheckMate‐77T [50], NeoTorch [55], Rationale‐315 [55], CheckMate‐816 [57], NADIM [58, 59]) it always contained a chemotherapy component. Of note, however, also in the adjuvant trials, most patients received (optional or mandatory) chemotherapy in these studies, too. The results were quite heterogenous, ranging from numerical advantages for males in the CheckMate‐77 T, AEGEAN, NeoTorch and Rationale‐315, the latter two with very few female patients, to a numerical advantage for females in the PEARLS, IMpower010, and KEYNOTE‐671 trials as well as the small single arm NADIM and NADIM II trials. In the (formally negative) BR.31 study, a minimal numerical advantage for female patients was observed (HR for DFS 0.92 (0.75–1.14) in males, 0.83 (0.61–1.12) in females) [51]. Study settings and outcomes are summarized in Table 1.

## 8 NSCLC Trials: Conclusions

In summary, when considering specifically the immunotherapy trials for NSCLC, the clearest effect appears to be that independently of the exact immune checkpoint inhibitor, men derive a larger benefit from mono‐immune checkpoint inhibitors as first‐line therapy. Both in second and further lines and in chemo‐immunotherapy trials in the palliative situation, as well as in trials in the early disease setting, the picture is less clear, with some trials without differences in benefit and some trials favoring male or female sex, respectively. The reason may be that only the first‐line trials with mono ICIs for advanced disease were completely “pure” immunotherapy trials, whereas all other trials and settings involved chemotherapy in some way, with varying intervals between chemo‐ and immunotherapy components.

## 9 Sex Differences in Immunotherapy and Chemo‐Immunotherapy Trials for Other Cancers

Only a limited number of cancers in addition to NSCLC were shown to benefit from mono‐immunotherapies. Of note, for most other cancers, the number of immunotherapy and/or chemo‐immunotherapy trials is more limited.

Interestingly, two independent trials with MSI‐high colorectal cancer both showed a potential larger benefit for males, in the case of the non‐randomized CheckMate 142 a higher overall response rate for males [60], and in the case of the randomized KEYNOTE‐177 an improved hazard ratio for overall survival for males (0.61 (95% CI 0.38–0.99) in males, 0.88 (95% CI 0.55–1.41) in females) [61]. Preliminary reports from the ATOMIC trial with MSI‐h colon cancer also indicate that both sexes benefit from chemo‐immunotherapy to a similar extent [62]. In a real‐world study comparing benefit from immune checkpoint inhibitors in MSI‐h colorectal cancer [63], the presence of BRAF mutations in addition to high MSI was associated with a worse PFS in response to ICI monotherapy in males compared to females, whereas this difference was not observed in the BRAF wt patients, patients treated with CTLA4/PD(L)1 antibody combinations or in patients not treated with immunotherapies. In exploratory analyses, the BRAF‐altered, MSI‐h tumors in males were enriched for an androgen signature and less presence of immune cells (memory B, activated NK, activated myeloid dendritic) in the TME.

In squamous cell carcinomas of the head and neck (HNSCC), a male advantage was observed in several trials where a checkpoint inhibitor was compared to chemotherapy (CheckMate 141, KEYNOTE‐040, KEYNOTE‐048 also including cetuximab, see details below) [64, 65, 66].

When urothelial cancers were treated with checkpoint inhibitors (KEYNOTE‐361 and KEYNOTE‐045 against chemotherapy, JAVELIN Bladder 100 as maintenance after chemo versus best supportive care, CheckMate 274 adjuvant versus placebo, KEYNOTE‐052 non‐randomized), the picture was overall very mixed, with no clear tendency towards either sex [67, 68, 69, 70, 71, 72].

In cancers of the upper gastrointestinal tract, that is, squamous cell cancer of the esophagus and/or gastric cancer, no clear advantage emerged for either sex, either, whether with immunotherapy alone (nivolumab/ipilimumab arm of CheckMate 648, Rationale‐302, ATTRACTION‐2 compared to placebo, pembrolizumab arm of KEYNOTE‐062, or chemo‐immunotherapy; e.g., nivolumab/chemotherapy arm of CheckMate 648, KEYNOTE‐590, KEYNOTE‐811 with additional trastuzumab, ATTRACTION‐4, KEYNOTE‐859) [73, 74, 75, 76, 77, 78, 79, 80, 81].

In melanoma, chemotherapy with Dacarbazine was used historically, but did not yield strong responses. Therefore, in both palliative and adjuvant melanoma trials, immune checkpoint inhibitors were used as monotherapies including combined checkpoint inhibitors, but not as chemotherapy combinations. For this cancer type, a strong tendency to a male advantage was also seen in adjuvant trials (KEYNOTE‐054, CheckMate 76K, KEYNOTE‐716) [82, 83, 84]. Finally, in other cancers (HCC, RCC) checkpoint inhibitors were mostly compared to targeted therapies such as sorafenib or sunitinib instead of chemotherapies, and often also combined with targeted therapies (e.g., RATIONALE‐301, CheckMate 459, HIMALAYA for HCC, CheckMate 025, JAVELIN Renal 101, KEYNOTE‐581 for RCC), and the results were quite heterogeneous with respect to a potential sex bias [85, 86, 87, 88, 89, 90].

## 10 Chemotherapy, the Immune System, and Potential Synergies

As already discussed above, it was suggested that while male cancer patients might benefit more from immune checkpoint inhibitors on their own compared to chemotherapy, female cancer patients might benefit more from the addition of an immune checkpoint inhibitor to chemotherapy compared to chemotherapy alone. Various chemotherapeutic agents were reported to be able to induce immunogenic cell death (recently reviewed in [91]) and shifting the transcriptional profile of tumors towards better responses to checkpoint inhibitors [92], thereby enhancing the activity of the immune system to recognize tumors and synergizing with immunotherapies, but this may also vary between the sexes and by treatment regimen.

In an analysis of the outcomes within the ECOG E1594 trial, the authors investigated whether there were sex‐specific differences in survival between male and female NSCLC patients that received one of four platinum‐based chemotherapy regimens [92]. While male and female patients had similar response rates (19%), women had a statistically significantly longer median survival (OS 9.2 months in women versus 7.3 months in men, PFS 3.8 months in women versus 3.5 months in men), despite a higher toxicity in female patients. Similar results with a trend to a longer survival in females were obtained in the TAX326 trial [93] as described in Wakelee et al., 2006. However, as these trials compared different chemotherapy regimens rather than chemotherapy versus placebo or a non‐chemotherapy, it remains unclear whether female NSCLC patients do derive a larger benefit from chemotherapy alone than men, or if other factors may contribute to their longer survival.

As opposed to most checkpoint inhibitor trials, in head and neck squamous cell carcinomas (HNSCCs), the KEYNOTE‐048 trial [66] had three arms where pembrolizumab with or without chemotherapy was compared to chemotherapy plus cetuximab in a cohort that did not require PD‐L1 positivity. Direct comparison between the pembrolizumab monotherapy and pembrolizumab plus chemotherapy arm was not reported. A moderate male advantage in OS was observed when pembrolizumab was compared to chemotherapy and cetuximab in the total population at the final analysis (HR 0.80 (0.66–0.97) for males, 0.89 (0.5–1.41) for females). This difference was also seen in the PD‐L1 high population (CPS ≥ 20, HR 0.60 (0.44–0.82) in males versus 0.70 (0.35–1.42) in females) as well as in the PD‐L1 low population (CPS ≥ 1, HR 0.71 (0.57–0.87) in males, 0.85 (0.52–1.37) in females). When pembrolizumab was combined with chemotherapy, the outcome showed somewhat different trends: At the final analysis, women had a slight numerical survival advantage (HR 0.72 (0.59–0.89) in males versus 0.67 (0.42–0.1.06) in females in the total population) and a similar advantage in the CPS ≥ 1 population (HR 0.66 (0.53–0.83) in males, 0.59 (0.36–0.96) in females), whereas males still retained a numerical advantage in the CPS ≥ 20 population (HR 0.57 (0.41–0.80) for males, 0.63 (0.32–1.23) in females). The different HRs are summarized in Table 2 for easy comparison. Although the “experimental” groups are obviously not the same, the comparator is, and the general study setting is the same for all patients. Therefore, an indirect comparison of the deviation of the two pembrolizumab arms to the same standard arm is likely to be a better comparison than that of independent trials. The data seem to suggest that pembrolizumab monotherapy efficacy increases with increasing PD‐L1 in a similar manner, but starting from a higher level in males. Combination treatment appears to increase benefit particularly in PD‐L1 low tumors for males. Females seem to benefit from chemo‐immunotherapy at all PD‐L1 levels, although the added benefit from chemotherapy is smaller in PD‐L1 high tumors. This would indeed suggest that both sexes benefit from chemo‐immunotherapy for PD‐L1 low or ‐negative tumors, whereas females benefit more from chemo‐immunotherapies overall, and even when their tumors are highly PD‐L1 positive. These findings are broadly in line with the results from between‐trial comparisons for other cancer entities described above.

**TABLE 2:** Summary of the key results of the KEYNOTE‐048 trial with squamous cell carcinomas of the head and neck.

	Pembrolizumab (vs. chemotherapy/cetuximab), hazard ratio for overall survival		Pembrolizumab + chemotherapy (vs. chemotherapy/cetuximab), hazard ratio for overall survival	
Sex	Male	Female	Male	Female
Total population	0.80	0.89	0.72	0.67
Combined positive score ≥ 1	0.71	0.85	0.66	0.59
Combined positive score ≥ 20	0.60	0.70	0.57	0.63

In the KEYNOTE‐062 study, an ICI‐chemo‐combination as well as an immune checkpoint inhibitor combination was compared with chemotherapy alone for the treatment of gastric cancer [73]. Both sexes appeared to derive benefit from the addition of the checkpoint inhibitor with a trend to more benefit for female patients with higher CPS scores (HR for OS in the PD‐L1 CPS ≥ 1 population 0.90 (0.63–1.27) in females, 0.88 (0.70–1.11) in males, in the CPS ≥ 10 population 0.56 (0.30–1.03) in females, 0.73 (0.48–1.10) in males). When chemo‐immunotherapy was compared to immunotherapy in this setting, no substantial sex differences were observed (HR for OS in the total population, 0.89 (0.62–1.29) in females, 0.84 (0.67–1.05) in males).

It was suggested that chemo‐immunotherapies might work independently of established biomarkers such as PD‐L1 expression at baseline and high mutational load. Indeed, to our knowledge, no predictive biomarkers or tests for chemo‐immunotherapies have entered routine clinical practice and/or been assigned as companion diagnostics for such therapies, although candidate biomarkers have been reported in first studies. In one study focusing on neoadjuvant chemotherapy for NSCLC, for instance, the authors reported an association of pathological response with significantly higher pre‐treatment squamous cell carcinoma antigen (SCCA) levels as well as a significantly lower post‐treatment platelet‐lymphocyte ratio (PLR) [94]. Other blood‐ or TME‐based potential markers were also reported [95, 96, 97], but none of them have been prospectively validated so far.

## 11 Considerations Regarding Evaluation Metrics

Of note, most of the studies regarding sex differences in immunotherapy response presented hazard ratios when comparing the outcome for male and female cancer patients, thus answering the question whether men or women benefited relatively more from the immunotherapy‐based treatment relative to a selected “standard” treatment. This standard treatment often comprised chemotherapies with different regimens, but could also be a targeted therapy, for example, sunitinib (JAVELIN Renal 101, [90]) or everolimus (CheckMate 025, [88]) in the case of renal cell carcinomas (RCC), or sorafenib in case of hepatocellular carcinoma (HCC). Such comparisons on their own are difficult to interpret, as a larger “delta” in benefit may arise from a better response to the experimental treatment, but also from an inferior response to the standard treatment. Of note, a hazard ratio of 1 does not mean that the experimental treatment conveys no benefit for the analyzed patient group, but merely that it does not convey a larger benefit than the standard treatment. Most importantly, even if an experimental treatment conveys less benefit in one patient group than in another, it is still possible (and indeed even likely) that the overall outcome of the experimental treatment is better for both groups. This means, for instance, that even if the HR for women and ICI therapy is not as low as that for men, women can still benefit from replacing chemotherapy with immunotherapy, albeit to a lesser extent, and vice versa regarding chemo‐immunotherapies. Ideally, one would therefore also have to look at the response rates and the survival per se, to see which treatment actually yields the best outcome in every group in absolute terms. In this context, it may also be necessary to define the metrics that are best suited to evaluate benefit from immunotherapies. Somewhat unexpectedly, for instance, Pala et al. [98] reported a good correlation between progression free‐ and overall survival for male, but not for female patients receiving immunotherapies, which might contribute to explaining why different studies, but also different meta‐analyses have come to different conclusions. On a side note, the underrepresentation of female patients in almost all studies means that it is harder to show statistically significant changes or benefit, as error bars and confidence intervals usually widen with smaller group size. Moreover, as illustrated by the studies described above, the confidence intervals of HRs for the male and female subgroups usually overlap. Therefore, statements such as “a statistically significant benefit was only detected in males” and “one sex benefits more than the other” should be interpreted with caution. To avoid such issues in the future, clinical trials should encompass sufficient numbers of male and/or female patients to robustly investigate the benefit of experimental treatments in each sex.

## 12 Differential Response to Immunotherapies: Biomarkers and Confounders

If differences between the sexes in immunotherapy responses can be confirmed, the question arises whether this is an independent “intrinsic” effect or whether it could also be explained by “confounding” variables associated with sex that also influence the outcome of immunotherapies. In general, especially UV and cigarette smoke are known to greatly increase the risk of genetic alterations. More changes acquired in tumor relative to normal tissue, such as a high mutational burden and particularly microsatellite instability (MSI)/a mismatch repair defect (dMMR) in the tumor, are associated with an increased risk and risk of progression, but also with a better response to immunotherapy [99, 100, 101], recently also reviewed in [102]. Conversely, some, but not all driver mutations may decrease responsiveness to immunotherapy on their own, as described for EGFR and ALK alterations in NSCLC [103] (see below). Regarding treatment with EGFR and ALK inhibitors themselves, no sex‐specific differences were observed for Alk inhibition, but some studies suggest that women might respond better to EGFR inhibitors [104, 105].

Taking NSCLC as an example, it is known that smoking patterns differ between men and women to different extents, with an additional impact of ethnicity/country of origin or country of residence. Smoking patterns, in turn, not only influence the incidence of lung cancer, but also the histology and features of the resulting tumors. The smoking of filterless cigarettes, for instance, was associated with squamous cell carcinomas, which occurred much more often in male smokers [106]. Adenocarcinomas occur more often in females and non‐smokers and are associated with higher incidence of driver mutations and a lower tumor mutational burden, the latter two having been shown to be negative predictive factors for immunotherapies [19]. One aspect related to this, which might also be considered a potential confounder, is the clearer association of female sex and independently that of adenocarcinoma histology with exposure to second‐hand smoke in never‐smokers, as described in a recent meta‐analysis [107].

KRAS G12C mutations, which occur more frequently in smokers, were described to enhance response to checkpoint inhibitors [108, 109]. In an attempt to identify more biomarkers associated with the response to PD‐1 inhibitors, the authors of one very recent study profiled circulating cytokines and chemokines in NSCLC patients and analyzed the results also with a view on sex‐specific differences [110]. They found that while some plasma cytokine levels (CCL5/RANTES) were associated with response in both sexes, others were associated with response in either males (CXCL5) or females (CXCL10).

Regarding the potential impact of sex hormones on lung cancer incidence and prognosis, two comparisons might be especially elucidating: comparisons between females and males and comparisons between premenopausal and postmenopausal women. Whereas a large body of research exists for the latter comparison for “women's cancer”, most prominently for breast cancer, little evidence can be found regarding other cancer entities. One study published in 2003 and encompassing almost 15.000 female NSCLC patients found that while premenopausal women were generally diagnosed at more advanced stages, their prognosis was better than that of postmenopausal women when adjusted for covariates such as tumor stage and histology, which might suggest a positive impact of higher estrogen and/or progesterone levels on lung cancer prognosis [111]. Another study showed that lung cancer incidence in young women exceeds that in young men, despite a similar smoking prevalence [112]. In one study, the presence of estrogen and/or estrogen receptors correlated with worse outcomes [113].

Studies on hormone replacement therapy (HRT) might also yield information on the impact of the respective hormones on lung cancer risk and prognosis. Potential associations were investigated in several large studies (e.g., the Nurses' Health Study, the Women's Health Initiative, The Prostate, Lung and Ovarian Cancer Screening Trial or the vitamins and lifestyle study), yielding conflicting results, ranging from lowered to increased lung cancer risk through HRT and no influence versus a detrimental influence on prognosis, with a trend towards a risk reduction especially through estrogen alone [114, 115, 116, 117]. At least in part, some of these differences might be explained by different study settings, for example, different HRT protocols, different durations of exposure, and different cohorts with respect to age, smoking status, etc. Overall, the context‐dependent impact of sex hormones on lung cancer risk and prognosis still remains to be clarified. Of note, as also discussed elsewhere in this review, the impact of sex on prognosis may also change over time depending on the available therapies and their (in‐)dependence on hormonal factors, for example, immunotherapy.

## 13 Sex Differences in Immune‐Related Adverse Events

Another aspect to consider is that immune‐related adverse events (irAEs) were reported in some studies to occur more often in females (e.g., [118] for melanoma patients), consistently with the overall higher baseline incidence of autoimmune diseases in women [119]. This higher incidence may generally be influenced by the dosage of immune‐related genes on the X chromosome [120], and were recently found to be influenced by (female‐specific) Xist ribonucleoproteins in a mouse study [121]. Interestingly, in the melanoma study mentioned above, the higher incidence of irAEs in females was similar in post‐ and premenopausal women, suggesting that this phenotype may also be hormone level independent. In another retrospective study [122], premenopausal women had slightly more irAEs, in particular endocrine irAEs, than postmenopausal women and substantially more irAEs than men (67% versus 60% and 46%, respectively).

While these findings overall suggest more potential harm for female cancer patients treated with immunotherapies, such a bias could also be advantageous given that response rates to immune checkpoint inhibitors might be higher in patients that develop immune‐related side effects. This was observed independently of cancer type, albeit with most analyzed studies conducted for melanoma or lung cancer [123, 124, 125]. Of note, high‐grade irAEs still had a positive impact on the response rate, but a detrimental impact on overall survival according to the meta‐analysis [124].

There is some heterogeneity and an associated knowledge gap; however, regarding the differential occurrence of irAEs in females versus males. In the study conducted by Quandt et al. [123], the rate of irAEs emerging under immune checkpoint inhibitor therapy was very similar between males and females. Specifically, irAEs of the skin or endocrine system were associated with better survival, whereas most other irAEs were rather associated with worse survival. Sex was not significantly associated with prognosis in this study, with a weak tendency for better outcomes in males. Similar results were obtained in another study from Thailand, with only marginally more females than males with irAEs [126]. In a systematic review and meta‐analysis of 16 trials and 4658 patients (MOUSEION‐07, [127]), only a non‐significant trend towards more irAEs in female patients was observed, and a high risk of bias was detected. In summary, there appears to be a certain predilection of females for more irAEs, but this potential sex disparity is less clear than that for autoimmune diseases occurring independently of immunotherapy.

For the latter, up to 80% of all patients are female [128], albeit with different distributions across different autoimmune diseases ranging from an almost equal distribution between sexes for inflammatory bowel diseases to almost 95% female cases for hypothyroidism. The authors consider it likely that these differences are mostly caused by x‐chromosomal factors. Overall, however, more research is required to elucidate the relative impact of sex hormones, X‐chromosomal genes and potentially other factors on the incidence of autoimmune disease and of adverse events in immune checkpoint inhibitor‐treated patients in detail.

## 14 Sex Differences in Immune Responses—Clinical Knowledge Gaps

The available clinical data so far may suggest that men may derive more benefit from mono‐immunotherapy and women may derive more benefit from chemo‐immunotherapy relative to chemotherapy. However, due to the relative nature of these comparisons, the heterogeneity in magnitude of treatment effects observed in clinical trials so far and due to the absence of randomized controlled trial results comparing immune checkpoint inhibitor therapy with and without chemotherapy, it has not been shown unambiguously that men benefit more from mono‐immunotherapy than from chemo‐immunotherapy and vice versa for women, and for which exact patient population for example, with respect to PD‐L1 levels or TMB might this be the case for. In a recent Norwegian real‐world study [129], in which 410 patients with NSCLC and 50% PD‐L1 or more were included, the authors observed a lower risk of early death in patients that received a chemo‐immunotherapy combination therapy and a longer median overall survival compared with pembrolizumab monotherapy (22.6 months versus 14.2 months). Interestingly, in subgroup analyses, especially female patients and patients with a high PD‐L1 level of 75% or more appeared to benefit from combination therapy. Whereas the survival of patients receiving pembrolizumab monotherapy was very similar between male and female patients, the survival of females was substantially improved with chemo‐immunotherapy compared to pembrolizumab monotherapy, in contrast to that of male patients, whose survival was only marginally better under combination therapy. Of note, more than half of the included patients were female. In another, larger retrospective analysis of 3086 NSCLC patients with high levels of PD‐L1 (50% or more and 90% or more, [130]), a modest survival benefit was observed in favor of chemo‐immunotherapy in the first 6 months of treatment, which is in line with observations from trials comparing immuno‐ and chemotherapy, where chemotherapy also appeared advantageous early on, possibly due to its ability to induce faster responses. However, although females comprised almost half of the analysis cohort, the authors did not present analyses comparing female and male patients, except for the observation that overall, male sex was associated with a worse prognosis within the first year of treatment. In a Japanese study restricted to older adults with 78% male patients [131], similar results were obtained, with no significant survival improvement through chemo‐immunotherapy, but higher adverse event rates. These results are in line with a good benefit from mono‐immunotherapies and no improvement through chemotherapy in male patients, as three quarters of the study population were male. Interestingly, even in the subgroup with lower PD‐L1 levels, no benefit from additional chemotherapy was observed. Again, in this study no sex‐specific outcome analyses were presented.

In a retrospective study performed by Jang et al., including 1369 elderly patients with advanced melanoma treated with a combination of nivolumab plus ipilimumab or a PD‐1 inhibitor alone, male patients exhibited more favorable outcomes when treated with combination therapy, with an overall mortality risk approximately half that of their female counterparts. All patients receiving combination therapy had previously received ipilimumab. The authors suggest that tumor mutations, along with age‐related alterations in the estrogen signaling pathway, may contribute to the differential ICI treatment responses observed between men and women with melanoma receiving combination therapy [132]. Interestingly, in this study, no differences in response were observed in male and female patients that were treated with PD‐1 antibodies, independently of ipilimumab pre‐treatment, which about 30% of the patients in this group had received.

In a very recent study focused on the receipt of immunotherapy versus chemo‐immunotherapy in NSCLC patients [133], the authors found that the proportion of patients with high PD‐L1 levels (50% or more) receiving checkpoint inhibitor monotherapies was decreasing steadily, whereas the proportion in patients with low or moderate PD‐L1 levels (1 to less than 50%) receiving mono‐immunotherapy with checkpoint inhibitors remained constant on a lower level. Female sex was positively associated with the use of mono‐ICI therapy (HR of 1.44). This may indicate that other factors than the results obtained in the studies described above, which would suggest a preferential usage of chemo‐immunotherapy in female patients, may influence the choice of treatment, for example, personal preferences with respect to potential side effects such as hair loss or the necessity to contribute to family care. Similar trends were also observed in other retrospective studies (e.g., [130]); however, the differences were not large.

**FIGURE 2:**
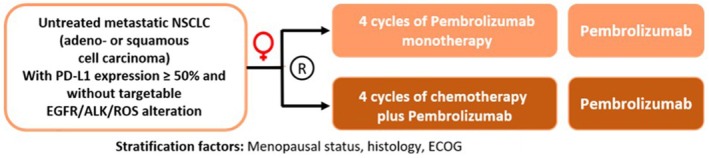
Outline of the IFEM trial. Note that the trial is female‐specific, as indicated by the female symbol.

Fortunately, first randomized trials are now starting to address the direct comparison between mono‐immunotherapy and chemo‐immunotherapy for both male and female NSCLC patients with high PD‐L1 levels. At the meeting of the European Society for Medical Oncology (ESMO) 2025, results from the Dutch PAULIEN trial (EudraCT 2024‐516581‐11‐00) were presented [134]. In this trial, NSCLC patients with a PD‐L1 level of 50% or higher received either pembrolizumab alone or in combination with chemotherapy. The trial was terminated after the first interim analysis, where no difference in response between the treatment arms after 6 weeks of treatment was seen. However, at that point, the ITT population comprised only 72 patients, essentially precluding further subgroup analyses by sex. Other currently running studies with this comparison are not powered to detect less than very striking differences between male and female participants. Our group has therefore acquired funding for the IFEM trial (Figure 2), a randomized phase III trial with more than 400 patients where female NSCLC patients will receive either mono‐immunotherapy or chemo‐immunotherapy, in order to compare directly whether they are actually undertreated by immunotherapy alone. Similar studies with male NSCLC patients should also be conducted to identify whether adding chemotherapy to immunotherapy might still provide some added benefit or whether it might even be detrimental, as the studies conducted so far seem to suggest. In our opinion, it will also be vital to include extensive translational research programs into these trials, so that potential mechanisms and confounding factors can be identified.

## 15 Potential Drivers of Sex Differences in Cancer and Immunotherapy Response

As described above, in the past few decades there has been a surge of interest in understanding sex disparities in cancer development and response to standard of care, including immunotherapies. In addition to cancer studies, several studies on autoimmune and infectious diseases have also clearly indicated that immune systems in males and females are distinct. The underlying causes can be varied: Sex‐based differences in immune profile are mostly attributed to sex hormones, sex chromosomes and/or epigenetics. The X chromosome contains many immune‐related genes, which may escape silencing, altering the dose of the products of the respective genes [120]. While sex hormones, most prominently estrogen and testosterone, are produced by males and females, the levels are radically different, with often more than 10 times the level of testosterone in men compared to women and a greatly fluctuating difference in estrogen levels depending on the female menstrual cycle for women of reproductive age, ranging from similar levels to 5 to 10 times the level of males in females [135, 136, 137]. Of note, after menopause, estrogen levels are quite similar in both sexes. Although there has been an exponential increase in studies to understand the contribution of sex and gender in cancer and response to cancer therapy, our understanding of the underlying mechanisms that dictate the distinct immune profiles in the tumor microenvironment is still lacking. Adding to this complexity is the age‐related fluctuations in hormones, environmental factors and contributions of lifestyle. Our understanding of the molecular and cellular mechanisms that guide the differences in immunity in general and specifically in antitumor immunity and response to immunotherapies in males and females is still rudimentary. Clinical meta‐analyses on different cancers have come to sometimes contradictory conclusions, necessitating further coordinated efforts in systematic studies that take into consideration additional confounding factors like therapy lines and combinations. Nevertheless, studies in cell culture and animal models as well as data derived from human samples have yielded first, albeit sometimes also heterogeneous, hints regarding the role of sex and possibly an influencing role of gender in the immune composition of tumors at baseline and the responses to cancer therapy in general and immunotherapy in particular. Of note, one reason for seemingly discrepant results regarding immune cell composition in the TME may be that the field of immunology, especially on T cell functional states and checkpoints, is very dynamic, including different markers and terminology.

In addition to sex hormones, the sex chromosomes play a major role in the sex‐based disparities in cancer incidence and progression. Loss of the Y chromosome (LoY) is a frequent, male‐specific somatic alteration across diverse cancer types that influences tumor pathogenesis and clinical outcome through interconnected genomic and immunologic mechanisms. In tumor cells, LoY is associated with adverse biological features including increased genomic instability, aneuploidy, elevated mutation and neoantigen burden, and disruption of Y‐linked tumor suppressor genes such as KDM5D, RPS4Y1, and SRY. This contributes to the increased aggressiveness of the disease as observed in esophageal adenocarcinoma, glioblastoma, uveal melanoma, and multiple myeloma [138, 139, 140, 141, 142]. These tumor‐intrinsic effects seem to intersect with immune dysregulation: experimental and pan‐cancer analyses demonstrate that LoY in cancer cells promotes immune evasion by impairing adaptive immune surveillance, and reshaping the tumor immune microenvironment [143, 144]. Importantly, LoY is not confined to malignant cells. Mosaic LoY in peripheral blood leukocytes is associated with increased cancer risk and reduced overall survival, indicating systemic immune consequences [145, 146]. Single‐cell analyses reveal particularly high frequencies of LoY in tumor‐infiltrating regulatory T lymphocytes, where it is linked to enhanced immunosuppressive phenotypes marked by an increased expression of checkpoint molecules such as PDCD1 and TIGIT [147]. While the prognostic impact of tumor‐cell LoY alone varies by cancer type and is limited in some settings [148], converging evidence indicates that concurrent LoY in both cancer cells and immune compartments confers the poorest outcomes, supporting a model in which intrinsic tumor aggressiveness and compromised antitumor immunity synergize to drive disease progression and influence response to immunotherapies in a context‐dependent manner [143, 144].

## 16 Potential Mechanisms Underlying Sex Bias in Cancer Incidence

**FIGURE 3:**
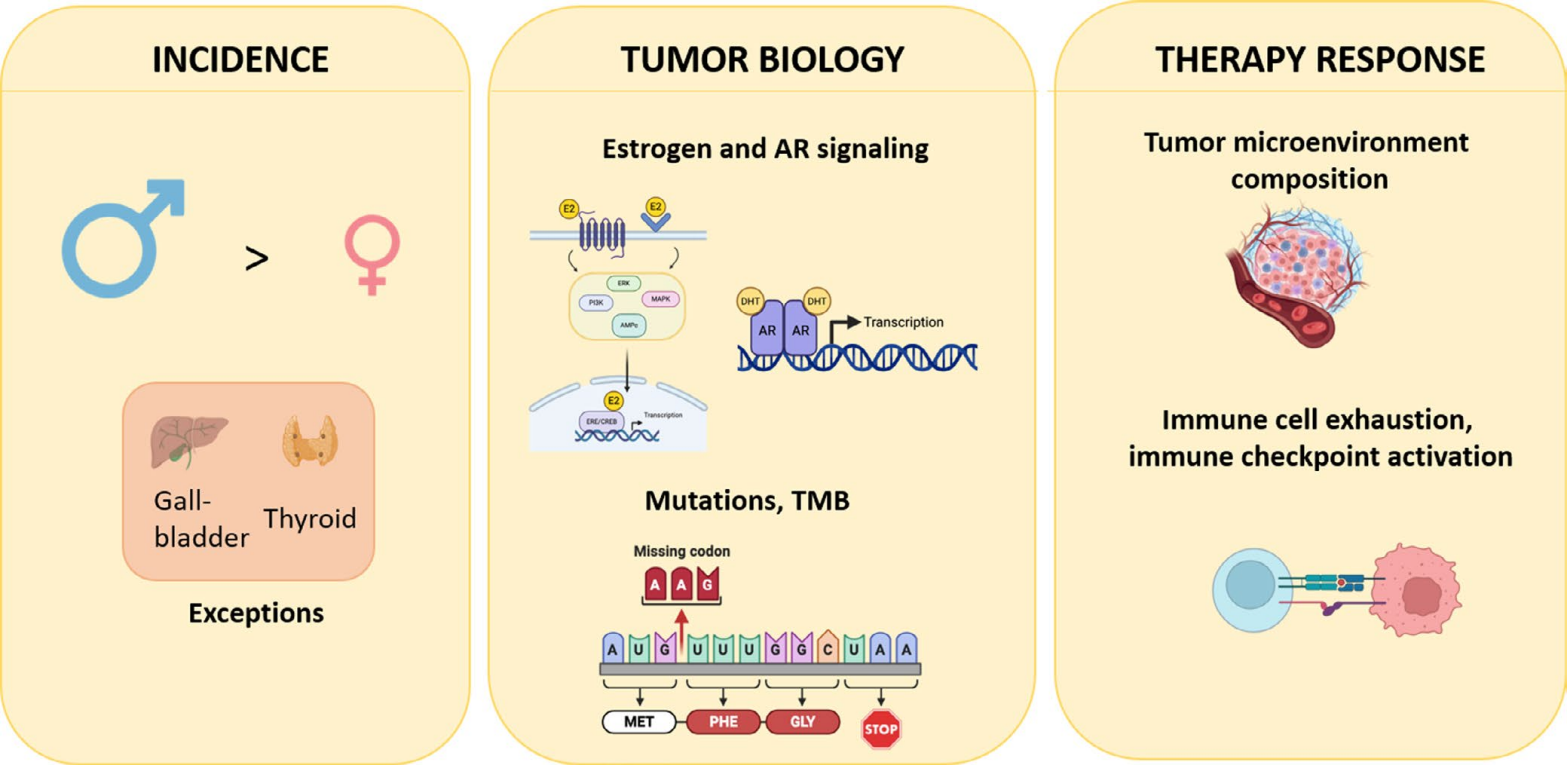
Impact of sex on cancer incidence, biology and therapy response. Cancer incidence is higher in men than in women, with the exceptions of gallbladder and thyroid tumors, which occur more frequently in women. The sex‐biased differences are partially attributed to estrogen and androgen receptor (AR) signaling, as well as differences in tumor mutational burden (TMB). In addition, tumor microenvironment composition, CD8^+^ T cell phenotype and expression of different inhibitory immune checkpoints have been shown to influence tumor behavior and response to therapy. Figure created using BioRender.

Cancer arises from a multifactorial interaction of genetic and environmental influences that vary between males and females [149, 150]. One study published that the higher incidence of gallbladder cancer in women might be associated with pancreaticobiliary malfunction (PBM) without biliary dilatation [151]. This study, involving 276 patients, showed that gallbladder cancer was found to occur frequently in individuals with PBM, and women had a higher risk than men. The higher incidence of thyroid cancer in women has been partly attributed to estrogen receptor–mediated signaling [152, 153]. Recent multi‐omics analyses comparing thyroid and breast cancers have identified hormone‐related pathways as a key shared feature of these malignancies. Importantly, estrogen and its receptors modulate fundamental biological processes in thyroid cancer cells, including proliferation, migration, and invasion [152]. Regarding lung cancer, as briefly discussed above, the higher incidence in males that is still observed in some parts of the world is thought to be driven by external influences, most prominently tobacco smoke. Lung epithelial cells express estrogen receptors and there is some evidence pointing to an influence of estrogens on lung cancer incidence as well, but the studies conducted so far have divergent results [154] (Figure 3).

## 17 Mechanisms Underlying Sex Differences in Baseline Tumor Biology and Therapy Response in Cancer Patients

Several factors that might be involved in the sex disparities regarding immune responses in human patients have been described, both at baseline and in response to immune checkpoint inhibitors.

For instance, androgens and androgen receptor (AR) signaling appear to play dual roles in thyroid cancer. Reduced AR expression in thyroid tissue has been linked to enhanced tumor progression, whereas AR overexpression has been associated with protective effects, including inhibition of epithelial‐to‐mesenchymal transition (EMT), restraint of cell proliferation, and reduction of PD‐L1 expression [153].

An additional work published in the same year presented findings in concordance with the previous study, while also delving into the molecular mechanisms underlying the observed sex‐related differences [155]. Shi et al. performed an integrated immunotherapy genomic analysis of 631 melanoma patients treated with immunotherapy, with the objective of elucidating sex‐related disparities in tumor response to ICI therapies. In this study, the authors observed a significantly higher tumor mutational burden (TMB) in male melanoma patients compared to female ones. Interestingly, analysis of significantly mutated genes (SMGs) in these patients revealed that three out of the 15 SMGs identified were associated with sex‐specific differences in the efficacy of ICI treatment. CFH, DGKG, and PPP6C mutations were linked to enhanced ICI response rates in males but not in female patients. Furthermore, the study demonstrated that males harboring CFH and DGKG mutations presented a significantly prolonged overall survival (OS) upon ICI treatment compared with male patients without these mutations, a difference that was not observed in female patients. Investigation of immune‐related mechanisms showed that effector memory CD8^+^ T cells and natural killer T (NKT) cells were significantly increased, while myeloid derived suppressor cells (MDSC) and regulatory T cells were decreased in male patients with CFH mutations. Thus, in melanoma patients, these three SMG mutations may serve as biomarkers for prognosis and immunotherapy efficacy in males, but not in females [155].

It was observed that female patients with LUSC exhibited significantly elevated levels of several biomarkers such as cytolytic immune activity by CD8^+^ T cells and NK cells in the tumor microenvironment. In the same study it was also found that female patients presented higher numbers of activated CD4^+^‐ and CD8^+^ ‐T cells. In addition, this study indicates that tumor cells in female patients show fewer chromosomal abnormalities, determined by aneuploidy scores, compared to those in male patients [156]. Somewhat surprisingly, female LUSC patients also showed increased levels of both inhibitory and activating checkpoint molecules.

A recent study has identified a mechanism underlying a male bias in CD8^+^ T cell progenitor exhaustion in basal cell carcinoma (BCC) and non–small cell lung cancer (NSCLC), which impacts antitumor treatment response and disease progression. In this study, AR was shown to directly regulate human *Tcf7* transcription by binding to its promoter, as demonstrated through ARE motif identification, promoter–reporter assays, and AR ChIP‐qPCR. Tcf7 encodes a transcription factor essential for maintaining the stem‐like and self‐renewing properties of CD8^+^ T cells, which are crucial for sustaining effective and long‐term antitumor immune responses [157] (also see below).

**FIGURE 4:**
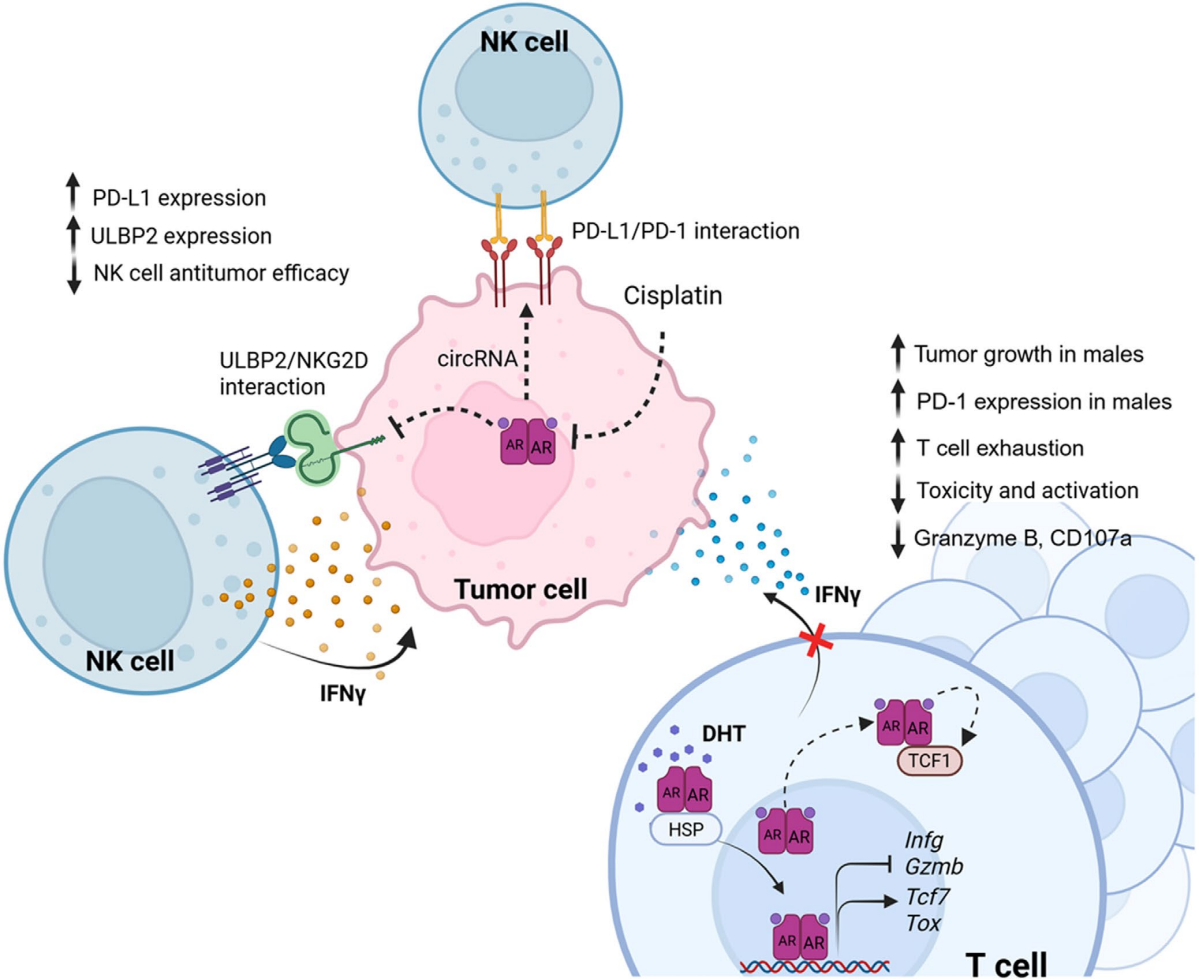
Androgen (receptor) shaping of the TME and antitumor immune responses. Testosterone signaling appears to downregulate effector cell activity, to induce immune checkpoint activation and an exhaustion phenotype. The impact on checkpoint inhibitor responses remains to be clarified. Figure created using BioRender; illustration by Dr. Ileana Hernandez Resendiz.

Conforti and colleagues presented data from several different human datasets suggesting that the response to immune checkpoint inhibitors in lung cancer patients differs between the sexes, with a key contributing factor being the composition of the tumor microenvironment. Specifically, they investigated the mechanisms mediating the anti‐tumor response in patients with early non‐small cell lung cancer (NSCLC). The female tumor microenvironment was characterized by an increased T‐cell dysfunction status, meaning higher levels of CD8^+^ and CD4^+^ T‐cells subpopulations with what they termed a pre‐exhausted phenotype. This was accompanied by elevated expression of inhibitory immune checkpoints, such as TIM3, VISTA, TNFRSF14, and TIGIT, which are relevant factors driving T‐cells exhaustion mechanisms and are associated in other studies with terminal rather than pre‐exhaustion. Conversely, male tumor samples were reported to exhibit a more “immune‐excluded” phenotype. These observations in human samples are somewhat contradictory to the results obtained in mice (see below), where several studies indicate that male mice harbor higher numbers of pre‐exhausted and possibly also exhausted cytotoxic T cells. The tumor microenvironment of NSCLC in women was also found to exhibit an increased infiltration of immunosuppressive cells, including CAFs, MDSCs, and Tregs, cell populations that contribute to inhibit effective antitumor immune responses [158] (Figure 4).

An additional study has reported sex‐biased stem cell‐like differentiation in human CD8^+^ T cells, with male donors exhibiting higher numbers of effector T cells with decreased expression of CD69 and fewer T memory stem cells compared to female donors. Interestingly, this male CD8^+^ T cell phenotype is linked to elevated intrinsic AR activity [159].

On the other hand, a study carried out in glioblastoma (GBM) has shown that CD8^+^ T cells in male patients are more exhausted than female CD8^+^ T cells. Male patients have also shown an improved responsiveness to anti‐PD‐1 treatment [160]. These results are in line with data obtained in melanoma patients, where the percentage of partially exhausted cytotoxic T lymphocytes (peCTLs), which was associated with better responses to single checkpoint inhibitor treatment, was lower in female patients and also in patients with liver metastasis [161]. In patients with lower peCTLs, response was greatly improved by combination immunotherapy also incorporating a CTLA‐4 directed antibody.

## 18 The Role of Androgen Receptor Signaling in Shaping the TME

Testosterone and the androgen receptor (AR) play a central role in the physiology and pathology affecting response to several diseases including cancer and response to therapy. AR signaling in the tumor microenvironment plays a major role through tumor cell intrinsic as well as immune cell intrinsic pathways. While the majority of the studies have focused on AR signaling in male cancers like prostate cancer, very little is known about how androgens and AR shape the infiltrating immune population in the TME.

Recent landmark studies spanning diverse types of human cancers including melanoma, bladder cancer, and hepatocellular carcinoma have revealed that AR activity is a critical driver of an immunosuppressive TME in males, with particularly profound effects on T cell and natural killer cell populations. The most striking and consistent finding that was described across several cancer types is AR‐induced CD8^+^ T cell exhaustion, resulting in impaired immunotherapy response leading to tumor progression according to the authors, especially in male mice (see Figure 4). Of note, as described above, in clinical trials, while male patients may have a worse prognosis overall, male patients appeared to benefit more than female patients from mono‐immunotherapies. Thus, further research is required to investigate whether a discrepancy between mice and humans with respect to sex bias in response to ICB exists.

### 18.1 Impact of AR Signaling on T Cells

The experimental studies focusing on non‐reproductive cancers show that AR promotes TCF1‐mediated stem‐like exhaustion in CD8+ T cells, in turn reducing their cytotoxic potential. In bladder cancer, androgen signaling promotes the transition of the CD8^+^ T cells from functional effector states to exhausted phenotypes, with androgen receptor directly binding to and regulating key transcription factors including TCF1/TCF7 (also see below), which governs T cell stemness and differentiation trajectories. Similar patterns emerged in renal carcinoma where androgens induced CD8^+^ T cell exhaustion and dysfunction in the male tumor microenvironment, while inhibition of AR reprograms the T cells towards an effector phenotype that appears to increase its response to PD‐1 blockade [157, 159, 162]. Androgen signaling seems to create transcriptional programs opposing antitumor immune responses with type I interferon pathways in regulating Tcf7 that favor immunosuppression over anti‐tumor immunity. In male mice, this results in T cells adopting a progenitor exhausted state (TCF1ᶫ°ʷTim3^−^) that progresses to terminal exhaustion, characterized by TOX expression and reduced effector function. Conversely, female murine T cells exhibited significant resistance to signals that induce exhaustion, all the while maintaining their effector function and superior anti‐tumor capacity compared to males. Mechanistically, androgen receptor‐mediated suppression of interferon‐responsive genes led to higher expression of exhaustion markers, creating a feed‐forward loop of immune dysfunction that fundamentally alters the tumor immune landscape.

Additional mechanisms contribute to this remarkable resistance of female T cells to exhaustion via X chromosomal factors. Lee et al. [160] demonstrated in gliomas that the T cell reprogramming can be attributed to AR as well as to Kdm6a, a histone demethylase encoded by an X‐linked gene and targets H3K27me3 that is important for T cell differentiation and effector function. In females, exhausted T cells have a higher expression of Kdm6a (UTX), contributing to increased cytokine production and maintenance of their functionality. This could indicate the importance of cell intrinsic as well as extrinsic factors that influence the sex disparities observed or different mechanisms predominating in a cancer‐specific manner. It is interesting to also note that Kdm6a mutations are found in various cancers and correlate with poor prognosis and that it is an X chromosome inactivation escape gene. Most interestingly, however, in this study, the authors report accelerated tumor growth in conjunction with a worse survival in untreated male mice, but a greater benefit from immune checkpoint inhibitors, which recapitulates the phenomenon frequently observed in trials with human lung cancer or melanoma patients. This was attributed by the authors to the higher number of progenitor exhausted T cells in the male TME, which are still able to be activated via immune checkpoint inhibition.

An additional mechanism operating in driving the male‐specific T cell activity was revealed through a comprehensive study across various cancers including melanoma, colon, lung, breast, and prostate cancers, where males consistently exhibit faster tumor growth and shorter survival than females [163]. Androgen receptor activation in CD8^+^ T cells directly upregulates USP18, a deubiquitinase that removes ubiquitin from TAK1, preventing TAK1 phosphorylation and subsequently suppressing NF‐κB signaling—a critical pathway for T cell activation and effector function. This AR‐USP18‐TAK1‐NF‐κB axis resulted in profound CD8^+^ T cell exhaustion, characterized by reduced IFNγ and TNFα production, increased expression of inhibitory receptors (PD‐1, TIM3, TIGIT), and impaired anti‐tumor immunity in males. Importantly, genetic ablation of androgen receptors in T cells or pharmacological androgen deprivation through orchiectomy or abiraterone treatment restored T cell function and decelerated tumor growth. The authors also report that reducing testosterone levels or testosterone signaling via AR significantly enhanced the efficacy of anti‐PD‐1 immunotherapy. However, it is perhaps noteworthy that while reducing testosterone (signaling) did indeed very noticeably decelerate tumor growth in mice, tumor growth was substantially reduced by PD‐L1‐directed checkpoint inhibitors in the presence or absence of high testosterone signaling. This was also the case in the study of Yang et al. [153]. In the study of Guan et al., survival outcomes under treatment with PD‐L1 inhibitors were better when testosterone signaling was inhibited [162]. Finally, Kwon et al. showed that tumors grew much faster in the presence than in the absence of testosterone, in which situation PD‐L1 inhibitors profoundly inhibited tumor growth [157]. Under testosterone suppression, tumors grew much more slowly, and PD‐L1 inhibitors hardly had an effect. In summary, the clearest and most reproducible effect in these studies was the accelerated tumor growth in the presence of high testosterone levels. This phenotype was dependent on immune cell‐ and most likely specifically CD8+ T cell function. Zhang et al. [163] also showed in human patients with non‐small cell lung cancer and melanoma that males with lower testosterone levels had better outcomes and higher CD8^+^ T cell infiltration in an analysis that was conducted independent of a specific treatment such as immunotherapy.

In certain cancer types like renal cell carcinoma (RCC) male malignant cells exhibit highly activated epithelial‐mesenchymal transition (EMT), angiogenesis, and transforming growth factor‐β (TGF‐β) pathways [164]. Interestingly, the androgen response scores show strong correlations to EMT and angiogenesis scores and critically, TGF‐β scores in males, suggesting TGF‐β signaling as a major downstream effector of androgen‐mediated tumor progression. An interesting paradox observed in this study was higher CD8^+^ T cell infiltration in the tumor microenvironment compared to females, while these T cells were predominantly exhausted and dysfunctional, with significantly higher levels of terminal exhaustion markers (PDCD1, LAG3, HAVCR2) and reduced cytotoxic function (decreased IFNγ, TNFα, and GrzmB production). Clinical validation in cancer patients revealed higher serum androgens also correlated significantly with increased CD8^+^PD1^+^ T cell percentages and worse prognosis in male RCC patients receiving immunotherapy. Therapeutically, androgen receptor inhibitors (enzalutamide) combined with anti‐PD1 therapy demonstrated synergistic effects, maximizing CD8^+^ T cell cytotoxicity while minimizing exhaustion, suggesting that the androgen‐TGF‐β axis represents a druggable pathway for enhancing immunotherapy efficacy in male RCC patients.

### 18.2 Impact of AR Signaling on NK Cells

While the studies described above have started to uncover AR‐mediated modulation of T cell function in the TME, very little is known about the role of AR signaling in the NK cells and other immune cells involved. A fundamental mechanism that the androgen receptor uses to modulate the NK cells seems to be suppressing IL‐12A expression, which compromises NK cell activity in hepatocellular carcinoma, affecting the intrinsic killing capacity of NK cells. The direct suppression thus establishes a baseline immunosuppressive environment that adversely affects males [165]. Combining AR targeting with sorafenib, an FDA approved multi‐kinase inhibitor for advanced HCC, and NK cell immunotherapy to enhance NK cell cytotoxicity, which enhances IL12A mediated NK cell function, would offer a multi‐modal treatment strategy for HCC. A complementary mechanism operates in HCC where AR transcriptionally suppresses ULBP2 (UL16‐binding protein 2), a critical NKG2D ligand essential for NK cell activation and tumor recognition, by directly binding to its promoter [165]. Interestingly, cisplatin enhances NK‐cell cytotoxicity in vitro and improves NK‐cell immunotherapy efficacy in vivo by suppressing AR through two mechanisms: increasing miR‐34a‐5p expression and promoting AR protein degradation via enhanced ubiquitination. The resulting AR suppression allows ULBP2 upregulation, significantly enhancing NK cell cytotoxicity against hepatocellular carcinoma cells. Thus, the chemotherapy backbone might play an important role in multimodal strategies aiming to increase NK cell anti‐tumor activity.

In bladder cancer cell lines, a sophisticated post‐transcriptional mechanism operates where androgen receptor signaling upregulates the circRNA (circ_0001005) which acts as a decoy for miR‐200a‐3p, which targets PD‐L1, increasing its expression, making them more susceptible to NK cell cytotoxicity [166]. Similarly, Tang et al. showed blunted NK cell activity in response to high dose androgen in prostate cancer cell lines [167]. High dose DHT/AR seems to regulate PD‐L1 expression via a complex non‐coding RNA mediated signaling involving circRNA, circFKBP5, and miR‐513a‐5p.

Similar to T cells, this sex hormone signaling in NK cells could influence their activity, proliferation, and effector functions. This direct NK cell‐intrinsic AR signaling is still unexplored.

### 18.3 Impact of AR Signaling on Macrophages and Dendritic Cells

Tumor‐associated macrophages (TAMs) can promote tumor growth by supporting tumor cell proliferation, reorganization of the extracellular matrix (ECM) facilitating invasion and promotion of angiogenesis. In addition, they can also promote expression of checkpoint proteins on themselves and on cancer cells [168], thus influencing therapy response. IL‐1β promotes immunosuppressive tumor‐associated macrophages (TAMs) and upregulates PD‐L1 in tumor cells, fostering angiogenesis, and ultimately leads to immune evasion [169]. IL‐1β is an androgen‐responsive target and can inhibit IL‐1β, but ADT during prostate cancer treatment adversely affects this and increases IL‐1β production in TAMs in the TME, inhibiting activation of cytotoxic T cells and initiating regulatory T cells [170]. A promising strategy in this case is usually a combination of ADT with ICB and by targeting IL‐1β using drugs like canakinumab. AR signaling in macrophages also increases their migratory and invasive phenotypes in prostate cancer via upregulation of Triggering Receptor Expressed on Myeloid cells‐1 (TREM‐1) receptor, activating its downstream cytokines that promote TAM differentiation in prostate cancer. Enhanced protumor activity of macrophages via AR signaling was also described in other cancers like breast cancer [171] and HCC [172]. Most of these studies point towards a negative correlation of AR signaling in the TAMs for their ability to control tumor growth. AR acts cell‐intrinsically in myeloid progenitors to promote monocyte differentiation. This affects numbers and possibly the functional phenotype of monocytes/macrophages [173].

AR signaling in dendritic cells (DCs) suppresses T‐cell priming efficiency during infection in males, but direct evidence of AR signaling in DCs in the TME is still lacking. But the impaired CD8+ priming observed in castrated male mice in a mouse colorectal cancer model might indicate the influence of AR on dendritic cells [159].

### 18.4 Conclusions and Consequences

Multiple studies have hence revealed significant sex‐based differences in cancer pathogenesis and immunotherapy response mediated via immune cell‐intrinsic as well as tumor cell‐intrinsic pathways. The mechanistic basis centers around androgen‐induced dysfunction of major immune populations including T cells and NK cells especially in males compared to females. Most promising therapeutic strategies from these studies involve combining androgen receptor antagonists with established chemotherapy and immune checkpoint blockade. However, more research is necessary to understand differences and similarities with respect to the impact of androgen (signaling) on immune cells in different cancer types.

## 19 Role of Estrogen Receptor Signaling in Shaping the TME

Unlike androgen receptor signaling that mainly promotes an immunosuppressive TME, estrogen receptor signaling is known to be more biphasic in a context and tumor‐specific manner. It is interesting to note that overall survival in patients with ER positive tumors is improved, whereas ERβ is often lost in breast cancers. ERβ interacts with E2 to upregulate Claudin‐6 (CLDN6) expression, which promotes autophagy and inhibits cancer cell migration and invasion [174]. Estrogen signaling also influences immune phenotypes of both adaptive and innate immune cells. Most of the studies describing this have mainly focused on diseases other than cancer. Estradiol through ERα promotes Treg cell proliferation by upregulating FOXP3 expression. ERα also increases IL‐17A production by TH17 cells. Nevertheless, recent studies have identified tumor cell‐extrinsic and immune cell‐intrinsic ER signaling to play a major role in altering the TME and ICB therapy response. Estrogen also plays a complex and context‐dependent role in regulating T cells and NK cells within the TME. Effects of estrogen vary significantly by cancer type, receptor subtype, and the hormonal status. In colorectal cancer, estrogen signaling enhances CD8+ T cell activation, with an increased cytotoxic activity in females. In females, immune responses were stronger, as indicated by increased expression of EOMES in CD8^+^ T cells, suggesting more active cytotoxic and effector T‐cell functions. In addition, B cells from female tumors more frequently reached a mature, antigen‐presenting (MHC‐I) state and exhibited enhanced predicted interactions with T cells—implying improved antigen presentation and B–T cell cooperation in females compared with males [175]. But most of the conclusions were made from single‐cell analysis. Similarly, estrogen depletion in liver metastases increases the recruitment and cytotoxicity of both NK cells and NKT cells, along with promoting CCL5+/CCR5+ CD8+ T cell infiltration [176]. Anobile et al. [177] demonstrated in non‐small cell lung cancer (NSCLC) that an autocrine 17‐β‐estradiol/ERα loop, driven by intratumoral aromatase and downstream EGFR‐AKT/ERK signaling, transcriptionally up‐regulates PD‐L1 (CD274) more strongly in females than in males, thereby predicting reduced response to anti‐PD‐1/PD‐L1 checkpoint blockade; notably, combining an aromatase inhibitor (letrozole) with ICI restored CD8^+^ T‐cell, NK‐cell, and Vγ9Vδ2 T‐cell antitumor activity in immune‐reconstituted PDX models [177]. Complementing this, other studies [178] show that ERβ in colorectal cancer suppresses NF‐κB and TNFα inflammatory axes in both epithelial and immune compartments, thereby reducing chronic inflammatory rewiring but also potentially limiting immune activation in the TME. Estrogen receptor‐α (ERα) signaling in the myeloid compartment plays a central role in enforcing an immunosuppressive tumor microenvironment. In melanoma models, ERα activation in tumor‐associated macrophages (TAMs) drives polarization towards an M2‐like phenotype, reduces intratumoral CD8^+^ T‐cell infiltration, increases exhaustion marker expression, and diminishes responsiveness to anti‐PD‐1 therapy; conversely, pharmacologic ER blockade with fulvestrant reprograms TAMs and restores checkpoint sensitivity [179]. Estrogen‐driven immunosuppression extends to other myeloid populations: in estrogen‐insensitive tumors such as the ID8‐Defb29/Vegf ovarian carcinoma model, estrogen signaling induces pathologic myelopoiesis, expands monocytic and granulocytic MDSCs, and enhances their suppressive activity, whereas oophorectomy delays tumor progression [180]. These findings support potential therapeutic benefits of ovarian hormone ablation or ER signaling blockade when combined with immune checkpoint inhibitors in ER^−^ breast cancer and other malignancies.

Beyond macrophages and MDSCs, sex hormones also regulate dendritic‐cell (DC) differentiation and antigen‐presentation capacity: in syngeneic melanoma and breast cancer models, both estrogens and androgens modulate DC infiltration and FOXO3‐dependent transcriptional programs, reshaping the cDC1 versus cDC2 subset composition that dictates effective CD8^+^ T‐cell priming [181]. Although a direct mechanistic link between ER signaling and ATG7/ULK1 (Atg1) induction in TAMs has not yet been established, autophagy is known to regulate myeloid antigen presentation—ATG7 deficiency impairs macrophage maturation and reduces MHC‐II expression [182]—and tumor‐cell autophagy promotes immune evasion and T‐cell dysfunction [183, 184]. Similarly, androgen receptor (AR) signaling contributes to tumor–myeloid crosstalk; in prostate cancer, AR activity in cancer‐stem‐like cells and TAMs enhances macrophage polarization, tumor progression, and resistance to androgen‐deprivation therapy [185]. Altogether, these findings reveal that sex‐hormone receptor pathways—via ER and AR—coordinate the behavior of macrophages, MDSCs, and DCs through both transcriptional and autophagy‐linked mechanisms, thereby shaping the immunosuppressive TME and determining the effectiveness of immune checkpoint blockade.

Overall, estrogen signaling demonstrates a clear biological duality. ERβ‐dominant contexts promote epithelial stabilization and tumor‐suppressive autophagy programs in breast cancer—such as CLDN6‐mediated beclin‐1 activation—and may help preserve innate lymphoid surveillance. In contrast, ERα‐dominant signaling across macrophages, dendritic cells, MDSCs, NK cells, and T cells fosters an immunosuppressive TME characterized by impaired cytotoxic lymphocyte activity, enhanced suppressive myeloid polarization, and diminished sensitivity to immune checkpoint blockade. This divergence underscores the therapeutic importance of receptor‐selective estrogen modulation to restore antitumor immunity.

## 20 Role of Progesterone in Shaping the TME

Progesterone plays a vital role in the female and male reproductive system as well as in mood regulation. Again, progesterone levels are higher in women before menopause, at least in the luteal phase (https://www.ucsfhealth.org/medical‐tests/serum‐progesterone). Its receptor is expressed in reproductive organs as well as in many immune cells [185, 186, 187]. Progesterone treatment or overexpression of PR inhibits immune responses in the murine mammary gland and increases the number of mammary gland tumors, mainly by decreased infiltration of immune cells including NKT cells, NK cells, and T cells [188]. Progesterone can also induce CD8+ T cell exhaustion and dampen their activation in the TME. The progesterone treatment increases expression of B7‐H4, an onco‐fetal immune tolerance checkpoint, in HR+ breast cancer cell lines. Thus, progesterone regulates immune compartmentalization both in the TME as well as the placenta via B7‐H4. In addition to NK and T cells, PR signaling can alter myeloid and TAM phenotypes, including macrophage polarization and reduce their pro‐inflammatory cytokine production and allergic asthma in murine models [189]. An inverse correlation between TAM density as well as tumor progression and PR status, but not ER status, was reported by Jiang et al. [190], indicating a potential relation between PR loss and disease progression and relative TAM infiltration in precancerous endometrial lesions in endometrial endometrioid adenocarcinoma. In mammary tumors, progesterone receptor signaling directly downregulates antigen processing and MHC class I surface expression, thereby reducing CD8+ T cells and promoting tumor evasion, implicating PR activity as a mechanism of immunosuppression in HR+ tumors suggesting targeting of the PR/progesterone axis may improve immunotherapy responses [191]. Our understanding of the role of PR in ICB response or response to immunotherapy is essentially limited to breast cancer. In EMT‐6 breast cancer cells implanted in mice, tumors with high PR showed diminished response to anti‐LAG3 therapy. The overall proportions of LAG3+ CD8+ T cells and LAG3+ Treg T cells were significantly reduced in PR overexpressing mouse tumors [192]. In addition, tumor fragments derived from patients' tumors with lesser PR signaling (< 20%) showed better response to anti‐LAG3 treatment in vitro, indicating adverse effects of PR in immunotherapy response. How progesterone might influence the response to CTLA‐4 and/or PD‐1/PD‐L1‐directed checkpoint inhibitors in cancers like NSCLC or melanoma is currently unknown. These findings highlight PR signaling as a potential barrier to effective immunotherapy, particularly in hormone receptor‐positive cancers.

## 21 Role of Gut Microbiota in Influencing the TME and Immunotherapy Response

In addition to hormone, chromosomal and environmental factors that influence health and disease are the resident commensal microbiota of the gut, which can significantly modulate immune responses. The microbiome can profoundly influence cancer pathogenesis and response to immunotherapy by modulating the immune microenvironment response and there is emerging interest in this field [193]. Recent studies have revealed intricate interplay between sex hormones, gut microbiota composition and immune responses, revealing fundamental differences between males and females, and their influence on cancer and immunotherapy outcomes [194, 195, 196, 197]. Lattanzi et al. found that young female mice with intact estrogen signaling exhibited a reduced tumor burden and more favorable immune profiles at baseline compared to males or estrogen‐deficient females in a CRC model, with this advantage disappearing in older female mice. Estrogen signaling plays a critical role in shaping the gut microbiome composition and fecal microbiota transplantation confirmed advantages of the female‐associated microbiome in anti‐tumor immunity. Wang et al. and Xi et al. identified that the interaction of commensals and hosts via microbiota‐derived metabolites such as bile acids, is highly sex‐biased, affecting inflammation and tumor progression. Importantly, as the dietary habits between the genders appear to differ [197], and dietary habits are known to influence the microbiome [198], gender in addition to sex may also have a modulating influence on the microbiome.

Key immunomodulatory mechanisms that are regulated by gut microbiome include modulation of immune cell activity including T cell activation and recruitment [199, 200, 201]. Beneficial gut bacteria like *Bifidobacterium* species can enhance dendritic cell (DC) activation and maturation, which is critical for CD8+ T cell priming and accumulation in the TME in a melanoma mouse model. Interestingly, this study [199] also observed differences in tumor growth rate in genetically similar C57BL/6 mice from two different mouse facilities, which differ in their gut microbiota, again pointing to their important role in regulating tumor growth and immunity. Consistently, *Lactobacillus paracasei* , identified from a larger screen of beneficial commensals that significantly increased sensitivity to anti‐PD‐1 therapy in colorectal cancer patients. Mechanistically, *L. paracasei* enhances T cell dependent antitumor immunity by triggering the expression of CXCL10 in the tumors enhancing T cell recruitment [199, 200, 201]. *Lactobacillus* can also have immune activating effects through cGAS/STING‐dependent type‐I interferon induction, influencing CD8+ T cell infiltration as observed in metastatic gastric cancer. Again, higher relative abundance of this bacteria correlated to higher microbial diversity, which in turn seems to contribute to better response to anti‐PD‐1 immunotherapy in human patients. Cross‐reactive antigen presentation and immune mimicry are critical to microbiome‐mediated immune enhancement, which is also significant in the case of tumor antigens. Fecal microbiota transplantation increases antigen‐presenting cell infiltration in both gut and tumor microenvironments, with upregulation of gene sets related to peptide presentation via MHC class I. In two clinical trials to test fecal microbiota transplantation (FMT), transplants from immunotherapy responders showed reinduction of anti‐PD‐1 immunotherapy responsiveness in anti‐PD‐1 refractory melanoma patients [202, 203].

The gut microbiome was described to influence cancer immunity through diverse metabolic pathways. Beneficial bacteria contribute to immunomodulatory effects through production of short‐chain fatty acids and via cobalamin pathways which support microbial community, in turn enhancing anti‐tumor immunity in melanoma patients [204]. In a landmark study, the authors showed that different bacterial species and specifically the microbial metabolite inosine from one of these strains augmented the response to immune checkpoint inhibitors in four different mouse models of colorectal cancer, bladder cancer, and melanoma via tumor‐targeting T cells. The effect of inosine was observed to be dependent on expression of adenosine A2A receptor on T cells, indicating a close interplay of host immune system and gut microbiota in cancer therapy response [205]. Several mechanistic studies in several different cancers, in preclinical and clinical settings, have recently identified signaling pathways like cGas‐STING‐IFN‐I pathway and TLR4 pathway, which were found to exhibit sex differences in other contexts [206, 207] as critical modulators of gut microbiome and host immune cell interactions, eventually reshaping the tumor microenvironment [208, 209, 210].

In summary, sex‐specific microbiome signatures, interacting with sex hormone signaling and the tumor microenvironment, represent a multifaceted axis influencing cancer initiation, progression and treatment response. Despite these advances and mechanistic understandings significant gaps remain. Most clinical trials and microbiome studies do not stratify data by sex and personalized immunotherapy approaches rarely incorporate microbiome profiling, let alone sex‐specific microbial data. Further research should integrate sex as a biological variable in microbiome‐cancer studies, develop microbial markers that are sex‐specific. In addition, these studies should explore microbiome target therapies tailored to hormonal and microbial profiles of patients, which could significantly improve immunotherapy outcomes.

## 22 Mechanistic Insights into Differences in Incidence of Immune‐Related Adverse Events

The reasons for the predilection of females for irAEs that was observed in many studies are under‐researched and thus poorly understood [211]. Nevertheless, first studies have pointed out some differences that might underlie this phenomenon. As summarized in a recent review article, pharmacokinetic studies indicated a slower clearance of PD‐1/PD‐L1 directed checkpoint antibodies [212] in females, which may be connected to differential PD‐1 expression between males and females [213] on the one hand and sex hormone‐dependent differences in antibody distribution, capture and catabolism [214]. In addition, it was reported that estrogen can upregulate the number and activity of regulatory T cells (Tregs) [215, 216]. These differences might impact cancer control as well as autoimmune events as “two sides of a coin”.

The described association of autoimmune diseases and the X chromosome, X‐linked long non‐coding RNAs like Xist and/or escape of immune‐related genes from inactivation on females might also play a role in the frequency of irAEs in women versus men treated with checkpoint inhibitors, but evidence for this is challenging to acquire and therefore largely lacking.

## 23 Conclusion and Outlook

In summary, evidence for a major impact of sex (and possibly also gender via indirect, lifestyle‐dependent mechanisms) on responses to and benefit from immunotherapies in NSCLC and other cancers is accumulating, both from clinical trials and from basic research. However, the evidence from clinical trials with regard to sex differences is still limited and heterogeneous, and basic researchers have even obtained seemingly contradictory results with respect to the influence of sex hormones on immune responses. This likely reflects the multiple influences that are already known to impact the response to immunotherapies, such as the gut microbiome, active or passive smoking and the associated tumor mutational burden, etc. Thus, large knowledge gaps remain regarding the optimization of immunotherapies for both sexes. In current medical practice, sex/gender is therefore still not a parameter that is routinely used to guide cancer therapy, and there is a high medical need to conduct large sex‐specific trials or sex‐specific analyses within large clinical trials in order to clarify unambiguously whether and how sex needs to be factored in as a clinical variable for therapy selection in NSCLC and possibly other cancers.

## Funding

This work was supported by Deutsche Krebshilfe, 70114428, 70112835 and 70113510; Hector Stiftung II; Deutsches Zentrum für Lungenforschung; European Health and Digital Executive Agency, 101183407; Deutsche Forschungsgemeinschaft, 1863/9‐1, 1863/8‐1; Dietmar Hopp Stiftung, EZN, 1DH221Z122.

## Conflicts of Interest

The authors declare no conflicts of interest.

## Data Availability

The authors have nothing to report.
